# Three-Dimensionally Printed Patient-Specific Surgical Plates Increase Accuracy of Oncologic Head and Neck Reconstruction Versus Conventional Surgical Plates: A Comparative Study

**DOI:** 10.1245/s10434-020-08732-y

**Published:** 2020-06-22

**Authors:** Wei-fa Yang, Wing Shan Choi, May Chun-Mei Wong, Warit Powcharoen, Wang-yong Zhu, James Kit-Hon Tsoi, Marco Chow, Ka-Wai Kwok, Yu-xiong Su

**Affiliations:** 1 Department of Oral and Maxillofacial Surgery, Faculty of Dentistry, The University of Hong Kong, Prince Philip Dental Hospital, Hong Kong SAR, China; 2 Dental Public Health, Faculty of Dentistry, The University of Hong Kong, Hong Kong SAR, China; 3 Oral and Maxillofacial Surgery, Faculty of Dentistry, Chiang Mai University, Chiang Mai, Thailand; 4 Applied Oral Sciences, Faculty of Dentistry, The University of Hong Kong, Hong Kong SAR, China; 5 Department of Mechanical Engineering, The University of Hong Kong, Hong Kong SAR, China

## Abstract

**Background:**

Surgeons are pursuing accurate head and neck reconstruction to enhance aesthetic and functional outcomes after oncologic resection. This study aimed to investigate whether accuracy of head and neck reconstruction is improved with the use of three-dimensionally (3D)-printed patient-specific surgical plates compared with conventional plates.

**Methods:**

In this comparative study, patients were prospectively recruited into the study group (3DJP16) with 3D-printed patient-specific surgical plates. The patients in control group with conventional surgical plates were from a historic cohort in the same unit. The primary end point of the study was the accuracy of head and neck reconstruction. The secondary end points were accuracy of osteotomy, intraoperative blood loss, total operative time, and length of hospital stay.

**Results:**

The study recruited of 33 patients, including 17 in the study group and 16 in the control group. The patients’ baseline characteristics were similar between the two groups. The absolute distance deviation of the maxilla or mandible was 1.5 ± 0.5 mm in the study group and 2.1 ± 0.7 mm in the control group [mean difference, − 0.7 mm; 95% confidence interval (CI) − 1.1 to − 0.3; *p* = 0.003], showing superior accuracy of reconstruction for the patients with 3D-printed patient-specific surgical plates. Improved accuracy of reconstruction also was detected in terms of bilateral mandibular angles and bone grafts. Concerning the secondary end points, the accuracy of the osteotomy was similar in the two groups. No difference was found regarding intraoperative blood loss, total operative time, or length of hospital stay.

**Conclusions:**

This is the first study to prove that compared with conventional plates, 3D-printed patient-specific surgical plates improve the accuracy of oncologic head and neck reconstruction.

**Electronic supplementary material:**

The online version of this article (10.1245/s10434-020-08732-y) contains supplementary material, which is available to authorized users.

Together with the development of microvascular surgery, surgeons are seeking more accurate head and neck reconstruction after oncologic resection to enhance aesthetic and functional outcomes. However, a main disadvantage of autologous bone flaps is mismatch in the shape of donor bone, which needs to be cut and trimmed to fit the defects and better restore the natural appearance. Much effort has been devoted to facilitating bone manipulations, and computer-assisted surgery (CAS) has emerged in the twenty-first century as a viable option.^1^

With CAS, surgeons make virtual plans on a computer that will be executed in the operating room to guide the precise harvest and placement of bone segments to repair defects. Bone segments can be finely navigated for optimal restoration of the original skeleton. In previous studies, various devices were developed to navigate bone segments according to virtual plans, including cutting templates, rapid prototype skull models, and surgical navigation systems.^1^ However, the missing link between bone navigation and accurate reconstruction is the plate fixation procedure.^2^

In conventional procedure, bone segments are fixed using commercial off-the-shelf titanium plates, which should be manually bent and twisted to fit the bone anatomy (Fig. 1). The manual contouring process often is tedious and technique-demanding, and can adversely affect the precise location of bone segments.^2^ Even worse, the repeated bending can even lead to poor fatigue performance of surgical plates.^3^ The disadvantages of conventional surgical plates underscore the importance of developing patient-specific surgical plates. Compared with conventional plates, patient-specific surgical plates are designed and manufactured in a three-dimensional (3D) structure that aligns with the individual bone contour. Unlike conventional surgical plates, which need to be contoured based on bone anatomy, patient-specific surgical plates can navigate the folding and precise placement of bone segments and are expected to improve the accuracy of reconstruction.^2^ Meanwhile, because patient-specific surgical plates need no bending, they can be used to optimize bone reconstruction in a more efficient and standardized manner.

**Fig. 1:**
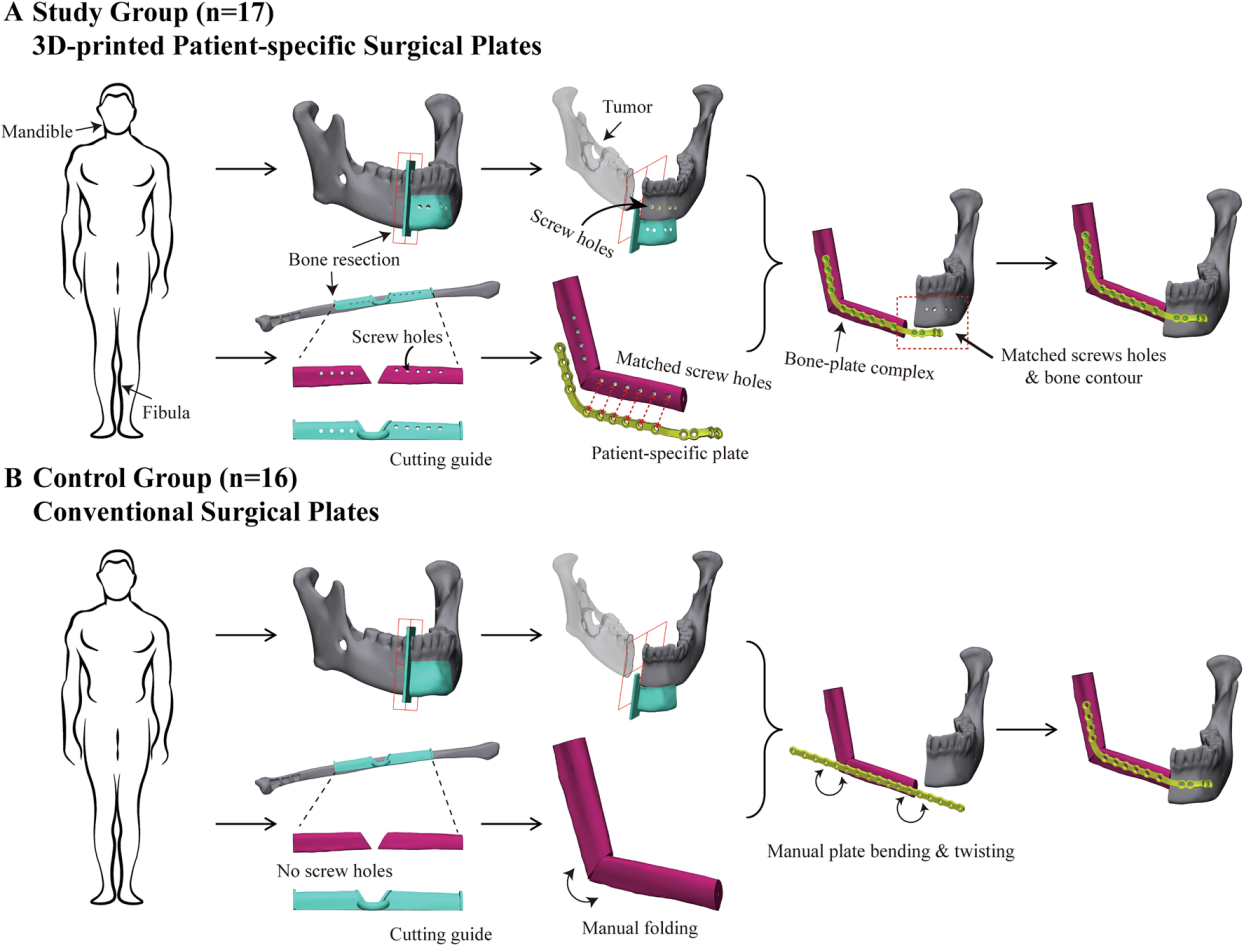
Study flowchart and working principles of combining three-dimensionally (3D)-printed patient-specific surgical plates with computer-assisted surgery (CAS) in head and neck reconstruction. **a** In the study group, patient-specific surgical plates guided the accurate folding and fixing of bone segments. Screw holes embedded in cutting guides correspond to those in the 3D-printed patient-specific surgical plates. **b** In the control group, bone segments were manually manipulated and stabilized using commercial off-the-shelf plates, which should be bent and twisted

In our previous studies, we successfully manufactured patient-specific titanium surgical plates with high precision through selective laser melting (SLM) technology.^2^ As a high-tech 3D printing technology, SLM fully melts titanium powders into complete entities in a layer-by-layer manner. The SLM technology enables fabrication of patient-specific surgical plates with tailored structures and good biomechanical properties.^4^^,^^5^ However, evidence concerning the application of three-dimensionally (3D)-printed patient-specific surgical plates in oncologic head and neck reconstruction still is lacking. Whether 3D-printed patient-specific surgical plates can improve the accuracy of reconstruction needs to be investigated.

To investigate the accuracy of head and neck reconstruction with 3D-printed patient-specific surgical plates, the current study quantitatively compared the patient-specific surgical plates with conventional plates. We hypothesized that the 3D-printed patient-specific surgical plates could improve the accuracy of head and neck reconstruction. Proof of this would constitute the highest level of evidence.

## Materials and Methods

### Study Design

The current study was designed to compare the reconstruction outcomes of 3D-printed patient-specific surgical plates with the outcomes of conventional plates in computer-assisted head and neck reconstruction. Patients receiving 3D-printed patient-specific surgical plates were recruited from the 3DJP16 clinical study (ClinicalTrials.gov Identifier: NCT03057223), an ongoing single-arm, prospective clinical trial studying the application of 3D-printed patient-specific surgical plates in head and neck reconstruction at the Queen Mary Hospital in Hong Kong. The initial nine cases were reported in our previous article.^2^

In the current study, the accuracy of reconstruction was analyzed by comparison with a retrospective control cohort that underwent CAS using conventional surgical plates. The patients in the control group had surgery performed by the same chief surgeon in the single unit between November 2014 and November 2017. All procedures were executed strictly according to the tenets of the Helsinki Declaration. This report has been prepared to meet all recommendations in the STROBE guideline.

### Participants

Patients were eligible for this study if they were older than 18 years, provided the signed informed consent form, had undergone computer-assisted maxillary or mandibular reconstruction using autologous bony free flaps after tumor resection, and agreed to comply with the follow-up procedures. Patients were excluded from the study if they were pregnant, unable to take computed tomography (CT) scans, had unstable or complicated systemic diseases that contradict the surgical process, had severe systemic diseases or conditions that compromise flap survival and the healing process, had reconstruction with nonvascular bone grafts, or had incomplete medical records including preoperative virtual plans and follow-up CT data.

### Computer-Assisted Surgery

The techniques of oncologic head and neck reconstruction using CAS in our unit have been described previously.^2^^,^^6^ Basically, CAS was composed of three main phases: the preoperative phase of virtual surgery and 3D printing of patient-specific devices, the intraoperative phase of precision-enhanced real surgery using patient-specific devices, and the postoperative phase of accuracy analysis.^1^ (Fig. 1).

In the preoperative phase, the virtual surgery was performed by surgeons using ProPlan CMF 2.0 software (Materialise, Leuven, Belgium). In ProPlan’s interactive interface, the patient’s CT data was initially segmented to construct 3D virtual models of the maxilla or mandible. Then, bone resection was performed in 3D virtual models for the en bloc removal of the tumor. Meanwhile, bone grafts were harvested from the iliac crest or fibula to repair defects and restore the normal appearance. Finally, the virtually reconstructed maxilla or mandible was used to design patient-specific devices for surgery.

### 3D Printing of Patient-Specific Devices

All patient-specific devices were designed in 3-Matic 13.0 (Materialise) using the “surgeon-dominated” approach.^6^ Cutting guides were designed by wrapping the bone surface to guide accurate bone resection. The cutting guides then were printed by Fused Deposition Manufacturing (FDM) using ULTEM 1010 or by Stereolithography using MED610 resin (Stratasys Ltd., Eden Prairie, MN, USA). Both ULTEM 1010 and MED610 are Food and Drug Administration (FDA)-cleared biocompatible materials suitable for high-temperature autoclaving.

Patient-specific surgical plates were designed by delineating a plate path on the bone surface and then placing the screw holes. Surgical plates were generated by the built-in command in 3-Matic. After that, surgical plates were fabricated by SLM using grade 2 titanium powder.

### Surgical Procedures

In the current study, all the patients in both groups had undergone CAS. During the surgery, patient-specific cutting guides enabled precise oncologic resection and bone flap osteotomy. However, in the control group, the arrangement of the bone segments was manipulated according to the mutual alignment of osteotomy planes, occlusion, and mandible contour through the surgeon’s experience and judgment call during the surgery. The bone segments were stabilized using commercial titanium surgical plates (DePuy Synthes, Raynham, MA, USA), which were bent manually, then fixed by screws^7^^–^10 (Fig. 1).

In the study group, except for the cutting guides, the patient-specific surgical plates were designed and fabricated to custom-fit the bone contour of the reconstructed maxilla or mandible.^6^ The screw holes embedded in the cutting guides corresponded to those in the 3D-printed patient-specific surgical plates and guided the position of the surgical plates and bone segments, thus facilitating the folding, positioning, and fixing of the bone segments in real surgery^2^ (Fig. 1). The standard perioperative management was similar in the two groups. Postoperative follow-up assessment was performed in the routine manner.

### Outcomes

**Fig. 2:**
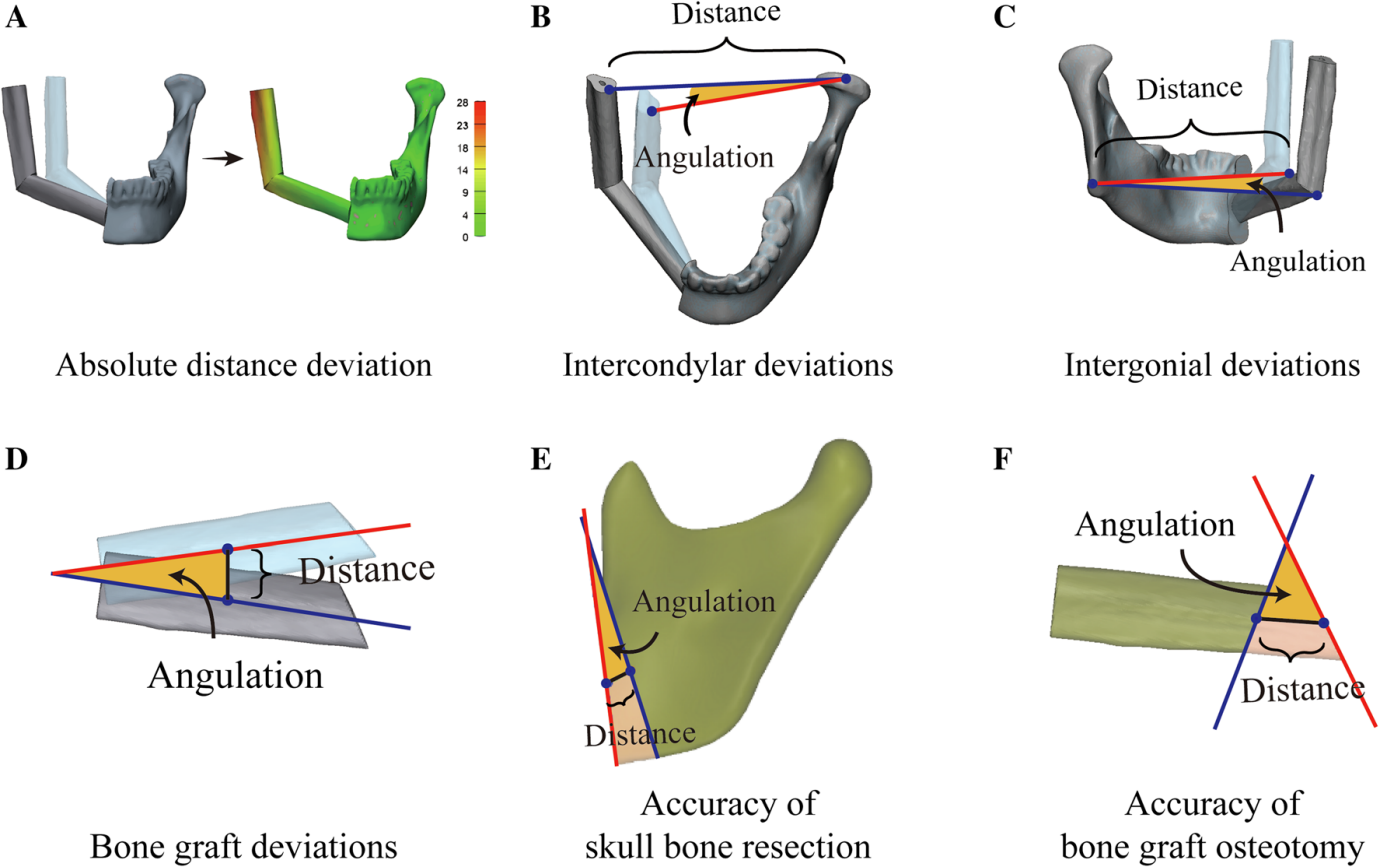
Accuracy outcome parameters of head and neck reconstruction. **a** Absolute distance deviation of the mandible. The postoperative mandible is repositioned to coincide with the preoperative virtually reconstructed mandible based on the side not treated by surgery. The absolute distance deviation of the mandible is measured by calculating the distance between the pre- and postoperative mandibles based on points in which green indicates small deviations, and red indicates large deviations. **b** Distance and angulation deviations of condylar heads. The intercondylar line is created by connecting the most superior points of the bilateral condylar heads. The distance deviation is defined as the difference in length of the pre- and postoperative intercondylar lines, and the angulation deviation is the angle formed by the pre- and postoperative intercondylar lines. **c** Distance and angulation deviations of mandibular angles. The intergonial line is created by connecting the most posterior inferior points of the bilateral mandibular angles. The distance deviation is defined as the difference in length of the pre- and postoperative intergonial lines, and the angulation deviation is the angle formed by the pre- and postoperative intergonial lines. **d** Distance and angulation deviations of reconstructed bone segments. The center point and central axis of each bone graft are generated. The distance deviation of bone graft is defined as the distance between the pre- and postoperative center points, and the angulation deviation is the angle between the pre- and postoperative central axes of the bone grafts. **e** Distance and angulation deviations of the skull bone resection. **f** Distance and angulation deviations of the bone graft osteotomy. In measuring the accuracy of the skull bone resection or the bone graft osteotomy, each bone segment is registered separately from the corresponding preoperative entity. Initially, the osteotomy planes are delineated, and the center points of each osteotomy plane are located. Then the angle formed by the pre- and postoperative osteotomy planes is defined as the angulation deviation of the osteotomy, whereas the distance deviation of the osteotomy refers to the distance between the center points of the pre- and postoperative osteotomy planes

The primary end point of the study was the accuracy of reconstruction, defined as the distance or angulation deviations of anatomic landmarks between the virtual plan and the actual surgical outcome. Various outcome parameters were established focusing on the entire maxilla or mandible, condylar head, mandibular angle, and bone grafts^2^^,^^11^ (Fig. 2). The postoperative skull model was repositioned to coincide with the preoperative virtually reconstructed skull based on the sides of maxilla or mandible not treated by surgery.^12^^,^^13^. The absolute distance deviation of the maxilla or mandible was measured by calculating the distance between pre- and postoperative models based on points, which was automatically calculated by the built-in function of the software.^2^^,^^9^^,^^10^

In evaluating the spatial displacement of the condylar head, an intercondylar line was created by connecting the most superior points of the bilateral condylar heads. The distance and angulation deviations of the condyle were derived by comparing the the pre- and postoperative intercondylar lines. The distance deviation was defined as the difference in length between the pre- and postoperative intercondylar lines, and the angulation deviation was the angle formed by the pre- and postoperative intercondylar lines.^14^^–^18 Similarly, we connected the most posterior inferior points of bilateral mandibular angles to form the intergonial line. The pre- and postoperative intergonial lines were compared to evaluate the distance and angulation deviations of the mandibular angle.^14^^–^20

In measuring the displacement of the transplanted bone grafts, the center point and central axis of each bone graft were generated. The distance deviation of the bone graft was defined as the distance between the pre- and postoperative center points, and the angulation deviation was the angle between the pre- and postoperative central axes of the bone grafts.^21^^–^23

The secondary end points of the study were accuracy of osteotomy, intraoperative blood loss, total operative time, and length of hospital stay. In measuring the accuracy of skull bone resection or bone graft osteotomy, each bone segment was separately registered to the corresponding preoperative entity based on designated landmarks (Fig. 2). We initially delineated the osteotomy planes and located the center points of each osteotomy plane. Then the angle formed by the pre- and postoperative osteotomy planes was defined as the angulation deviation of osteotomy, whereas the distance deviation of osteotomy referred to the distance between the center points of the pre- and postoperative osteotomy planes.^21^^–^23 Intraoperative blood loss, total operative time, and length of hospital stay also were assessed as secondary end points.

Postoperative adverse events associated with the surgical plates were summarized. All perioperative information was retrieved from the hospital’s medical database. The accuracy of both reconstruction and osteotomy was measured by two independent operators.

### Statistical Analysis

Dichotomous values were depicted as counts (*n*) with proportion and compared using the Chi square test or Fisher’s exact test as indicated. Continuous data are expressed as mean values with standard deviation (SD) for normally distributed data and compared using the independent-samples *t* test. Skewed data are expressed as medians with interquartile range (IQR) or range and compared using the Mann–Whitney *U* test. Mean differences of accuracy are accompanied with 95% confidence intervals (CIs). All tests and reported *p* values are two-sided. Statistical significance is set at a *p* value lower than 0.05. To account for the multiple measuring of the primary end point, the adjusted *p* value of 0.05/7 = 0.007 is used. All statistics were calculated using SPSS Statistics (version 22.0; SPSS, IBM Corporation, Chicago, IL, USA) and GraphPad Prism 5 (GraphPad Software Inc, San Diego, CA, USA).

## Results

### Clinical Demographic Characteristics

The study included 33 patients. Between December 2016 and July 2018, 19 patients underwent oncologic head and neck reconstruction using 3D-printed patient-specific surgical plates in the 3DJP16 clinical study. Two patients were excluded from the study because the one patient had reconstruction using nonvascular bone graft and the other patient with comorbidities of renal failure and poorly controlled diabetes experienced late-stage artery thrombosis and flap failure. Therefore, the study group included 17 patients for accuracy analysis who were managed with 3D-printed patient-specific surgical plates. The 17 cases involved 3 maxillary reconstructions and 14 mandibular reconstructions.

The control group comprised 19 patients who underwent CAS using conventional surgical plates between November 2014 and November 2017. Two patients were excluded from the analysis due to loss of preoperative virtual plans, and one patient was excluded due to lack of follow-up CT data. The study included 16 patients in the control group for accuracy analysis. These 16 cases involved 3 maxillary reconstructions and 13 mandibular reconstructions (Fig. 1).

The demographics and baseline characteristics were similar between the study and control groups including the distribution of age, sex, lesion types, maxillary or mandibular defects, and types of free bony flaps including fibula or iliac crest (Table 1). Four patients in the study group underwent “double-barrel” fibula flap mandibular reconstruction with the aid of 3D-printed patient-specific surgical plates compared with no patients in the control group. Eight patients in the study group received postoperative radiotherapy (RT) or concurrent chemoradiotherapy (CRT) compared with four patients in the control group (*p* = 0.19). The postoperative follow-up CT data were retrieved 1.8 ± 0.8 months after surgery in the study group and 17.3 ± 11.2 months after surgery in the control group.

**Table 1:** Demographics and baseline characteristics of the patients

Characteristic	Patient-specific surgical plates group (*n* = 17) *n* (%)	Control group (*n* = 16) *n* (%)	*p* Value
Age (years)	55.6 ± 14.4	55.4 ± 16.0	0.97^a^
Sex			0.71^b^
Male	4 (23.5)	5 (31.3)	
Female	13 (76.5)	11 (68.8)	
Lesion type			1.00^b^
Benign	3 (17.6)	4 (25.0)	
Malignant	12 (70.6)	11 (68.8)	
Other^c^	2 (11.8)	1 (6.3)	
pT3/T4 cancer^d^	6 (35.3)	6 (37.5)	0.90^e^
Defect site			1.00^b^
Maxilla	3 (17.6)	3 (18.8)	
Mandible	14 (82.4)	13 (81.3)	
Donor bone graft^f^			1.00^b^
Fibula	15 (88.2)	15 (93.8)	
Iliac crest	2 (11.8)	1 (6.3)	
Donor bone length (mm)	95.8 ± 38.7	88.2 ± 21.6	0.50^a^
No. of bone graft segments			0.89^g^
Median	2	2	
Range	1–4	1–3	
Plate thickness (mm)^h^			
Median	1.8	NA	
Range	0.8–2.0	NA	
Plate width (mm)^h^			
Median	4.0	NA	
Range	3.0–4.5	NA	
Postoperative RT/CRT	8 (47.1)	4 (25.0)	0.19^e^

*NA* not applicable, *RT* radiotherapy, *CRT* chemoradiotherapy

^a^This *p* value was calculated by means of the independent-samples *t* test

^b^This *p* value was calculated by means of Fisher’s exact test

^c^Other included secondary reconstruction and osteoradionecrosis

^d^Pathologic tumor category according to American Joint Committee on Cancer staging for head and neck cancer, 8th edition

^e^This *p* value was calculated by means of the Chi square test

^f^All defects were reconstructed using microvascular free flap

^g^This *p* value was calculated by means of the Mann–Whitney *U* test

^h^Plate thickness and width were based on the main body because the patient-specific surgical plate was not uniformly shaped

### Primary End Point

**Fig. 3:**
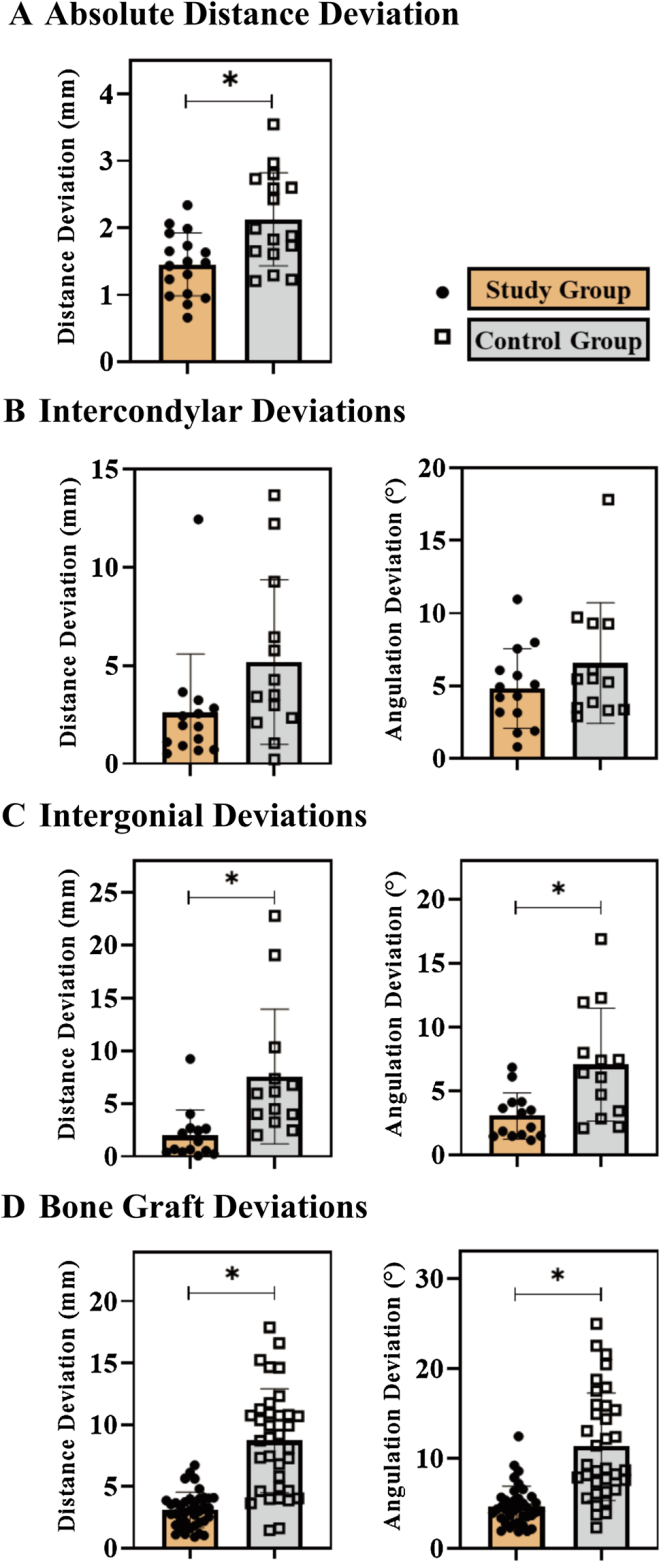
Accuracy results of head and neck reconstruction. **a** Absolute distance deviation of maxilla or mandible. **b** Distance and angulation deviations of condylar heads. **c** Distance and angulation deviations of mandibular angles. **d** Distance and angulation deviations of reconstructed bone segments. In all plots, error bars depict means and standard deviations. All statistical comparisons are performed using the independent-samples *t* test. All *p* values lower than 0.007 are indicated with an asterisk (*)

The primary end point, accuracy of reconstruction, was derived by comparing the postoperative maxilla or mandible with the preoperative virtual models. The inter-operator agreement was good to excellent (Table S1). The mean absolute distance deviation of the maxilla or mandible was 1.5 ± 0.5 mm in the study group and 2.1 ± 0.7 mm in the control group (mean difference, − 0.7 mm; 95% CI − 1.1 to − 0.3 mm; *p* = 0.003), showing superior accuracy of reconstruction outcomes in the study group with 3D-printed patient-specific surgical plates (Fig. 3a; Fig. S1).

For mandibular reconstruction, we aimed to evaluate the spatial displacement of bilateral condylar heads. The distance deviation between the pre- and postoperative bilateral condylar heads was lower in the study group with 3D-printed patient-specific surgical plates (2.6 ± 3.0 mm), but did not differ significantly from the control group (5.2 ± 4.2 mm) (mean difference − 2.6 mm; 95% CI − 5.5 to 0.3 mm; *p* = 0.076). Similarly, no significant difference was detected in the angulation deviation of the bilateral condylar heads (mean difference − 1.7°; 95% CI − 4.5° to 1.0°; *p* = 0.21) (Fig. 3b).

We further analyzed the deviations of bilateral mandibular angles. The 3D-printed patient-specific surgical plates significantly increased the accuracy of reconstruction in terms of distance deviation (mean difference − 5.6 mm; 95% CI − 9.4 to − 1.8 mm; *p* = 0.005) and angulation deviation (mean difference − 4.0°; 95% CI − 6.6 to − 1.4; *p* = 0.005) of intergonial lines compared with the control group (Fig. 3c).

The dislocation of transplanted bone segments was evaluated through the center point and central axis of each bone graft. The study group had 38 transplanted bone segments eligible for accuracy analysis compared with 35 in the control group. The distance deviation of the bone grafts was 3.1 ± 1.4 mm in the study group versus 8.7 ± 4.2 mm in the control group (mean difference − 5.6 mm; 95% CI − 7.0 to − 4.2 mm; *p* < 0.001). The angulation deviation also was significantly less in the study group than in the control group (mean difference − 6.6°; 95% CI − 8.7 to − 4.6; *p* < 0.001; Figs. 3d; Fig. S2).

### Secondary End Points

**Fig. 4:**
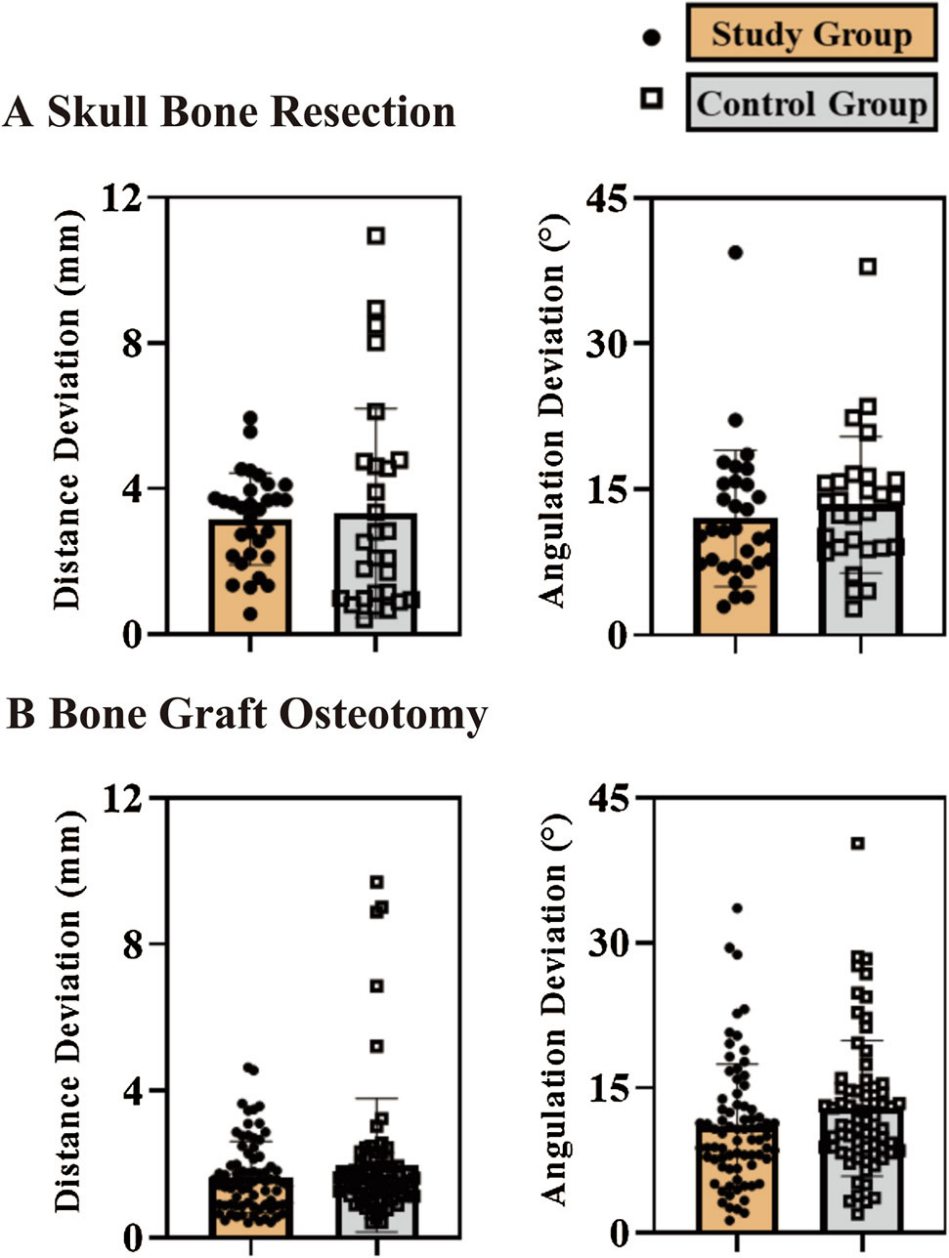
Accuracy of skull bone resection and bone graft osteotomy. **a** Distance and angulation deviations of skull bone resection. **b** Distance and angulation deviations of bone grafts osteotomy. In all plots, error bars depict means and standard deviations

We also evaluated the accuracy of osteotomy at both recipient and donor sites. The inter-operator agreement was fair to good (Table S1). No significant difference in accuracy of the osteotomy was detected between the study and control groups (Fig. 4; Fig. S3). As shown in Fig. 4a, the distance deviation of the maxilla or mandible resection was 3.2 ± 1.3 mm in the patients with 3D-printed patient-specific surgical plates and 3.3 ± 2.9 mm in the control group (*p* = 0.78). The angulation deviations did not differ significantly between the two groups (*p* = 0.45). As shown in Fig. 4b, the osteotomy of the bone grafts did not differ significantly between the study and control groups in terms of either distance deviation (*p* = 0.17) or angulation deviation (*p* = 0.10).

The intraoperative blood loss in the study group was similar to that in control group (*p* = 0.31) (Table 2). Likewise, the total operative time was comparable between the two groups (*p* = 0.99). The hospital stay was 15.1 ± 3.6 days in the 3D-printed patient-specific surgical plates group compared with 17.0 ± 5.7 days in the control group (*p* = 0.25). No major postoperative adverse events at recipient sites were identified in either group. Due to the small number of participants, statistical comparisons were not performed. Two patients in the control group underwent plate removal due to plate exposure compared with no patients in the study group.

**Table 2:** Intraoperative blood loss, total operative time, hospital stay, and postoperative adverse events at the recipient site

Characteristic	Patient-specific surgical plates group (*n* = 17)	Control group (*n* = 16)	*p* value
Estimated blood loss (ml)			0.31^a^
Median	800	900	
IQR	500–1000	500–1300	
Total operative time (min)			0.99^a^
Median	510	496	
IQR	453–602.5	446.8–630.5	
Mean hospital stay (days)	15.1 ± 3.6	17.0 ± 5.7	0.25^b^
Adverse events: *n* (%)			
Wound infection	2 (11.8)	3 (18.8)	
Plate breakage	1 (5.9)	0	
Plate removal	0	2 (12.5)	
Bone mal-/non-union	0	0	

*IQR* interquartile range

^a^This *p* value was calculated by means of the Mann–Whitney *U* test

^b^This *p* value was calculated by means of the independent-samples *t* test

**Fig. 5:**
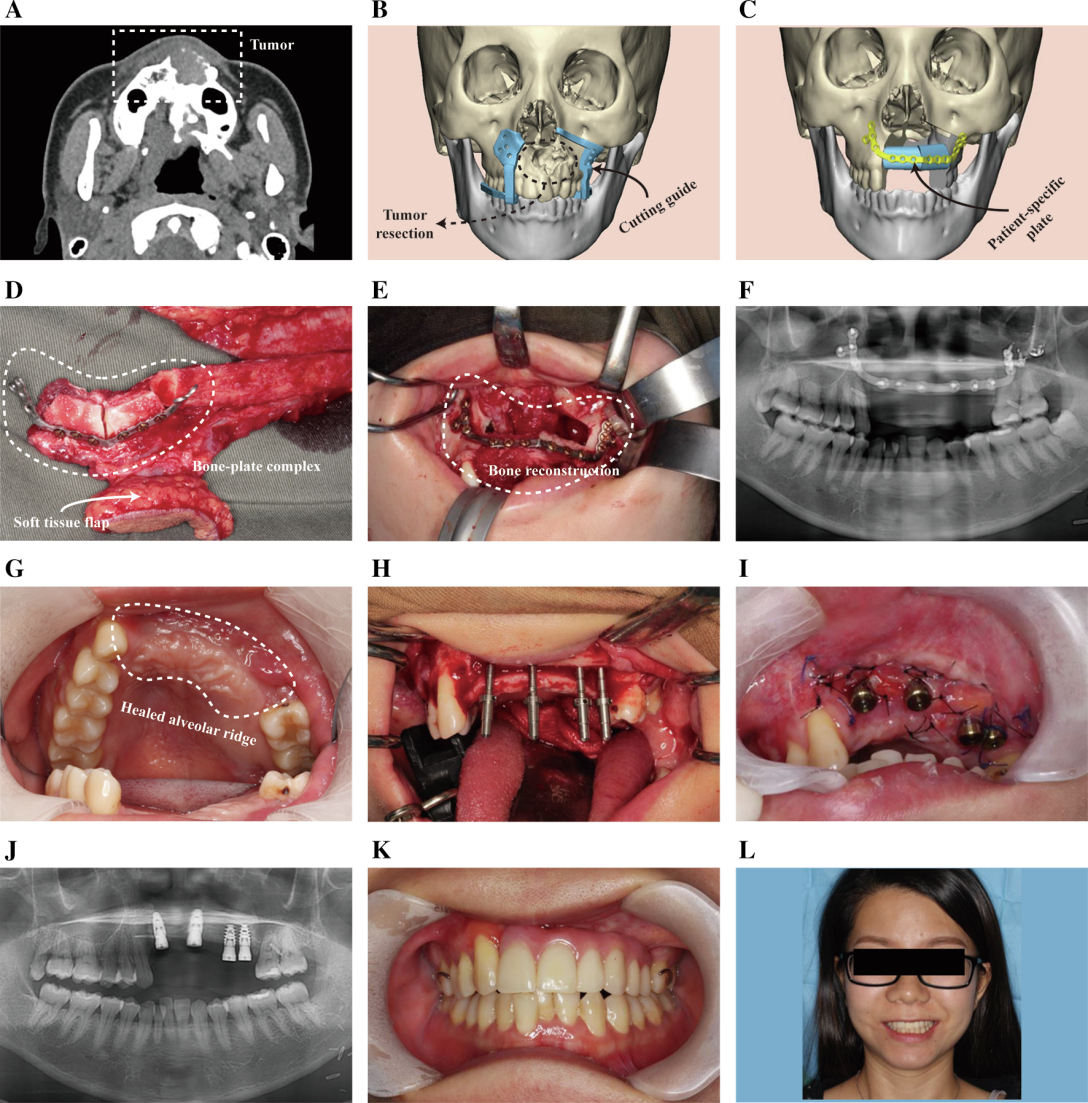
A 33-year-old woman with desmoplastic ameloblastoma in the anterior maxilla underwent surgery. **a** The preoperative computed tomography (CT) image indicates the destructive mass in the anterior maxilla. **b** A three-dimensional (3D) virtual model is used to delineate bone resection margins and design cutting guides. **c** The patient-specific surgical plate is designed to fix bone grafts. **d** The vascularized fibular flap is harvested, segmented, folded, and fixed in alignment with the patient-specific surgical plate. **e** The bone-plate complex is transferred to repair the defect site. **f** Postoperative OPG showing satisfactory bone healing. **g** Postoperative intraoral image showing healed alveolar ridge. **h** Dental implants placed in the transplanted fibula in the second stage. **i** Intraoral image showing the accurate position of implants as planned. **j** OPG showing satisfactory implant position and angulation. **k** Immediate loading of dental implants supports the removable partial denture in the anterior maxilla. Excellent occlusal relationship is achieved. **l** Satisfactory postoperative aesthetics

Bony union was achieved for all the bone segments in both groups, as shown on an orthopantomogram during the follow-up assessment. The occlusal functions were satisfactory for most of the patients with 3D-printed patient-specific surgical plates. For the patients with indications, dental implants were placed in the second stage to restore missing teeth, as illustrated in Fig. 5.

## Discussion

Our previous prospective single-arm pilot study explored the feasibility of using 3D-printed patient-specific surgical plates in head and neck reconstruction.^2^ Based on this, the current study further demonstrated that compared with conventional surgical plates, 3D-printed patient-specific surgical plates reduced the distance deviations of reconstructed maxilla or mandible, distance and angulation deviations of bilateral mandibular angles, and transplanted bone segments, thus leading to enhanced reconstruction accuracy. To the best of our knowledge, this is the first study to investigate the accuracy of outcomes from 3D-printed patient-specific surgical plates versus conventional surgical plates in head and neck reconstruction.

Computer technology enables CAS to study the accuracy of head and neck reconstruction by comparing postoperative reconstruction outcomes with preoperative virtual plans. However, no standard parameters exist to date.^11^ Different studies have tended to use different measurements, which severely limits the interpretation and comparison of results.^11^^,^^24^

The current study used a series of parameters to evaluate the accuracy of reconstruction systematically. The absolute distance deviation of the maxilla or mandible is the parameter most commonly used to evaluate the overall accuracy of reconstruction due to its simplicity and intuitive form for comparison.^2^

Considering the crucial functional roles of the mandible, we also assessed the displacement of condylar heads and mandibular angles, which are the most commonly evaluated landmarks according to the literature.^11^ Because the accuracy of condylar heads is affected bilaterally, we used the intercondylar line to connect the bilateral condylar heads to evaluate the overall accuracy of condylar heads. Meanwhile, it is simpler and more straightforward to understand and compare different studies by using the intercondylar line.^2^

Similarly, bilateral mandibular angles also were connected and evaluated using the intergonial line. In the Yu et al.^25^ study of mandibular reconstruction using CAS and navigation, the mean distance deviations of the condyle and gonion were respectively 9.3 ± 2.6 mm and 7.3 ± 2.5 mm, higher than those in our study. In the study of Bao et al.^13^ that analyzed pre-bent surgical plates, the distance deviation of the condyle was 6.0 ± 1.5 mm, and the distance deviation of the gonion was 5.8 ± 1.2 mm, comparable with those in our control group. However, if the pre-bent surgical plates were used together with screw-predesignated surgical guides, the distance deviations of the condyle and gonion were reduced to 2.6 ± 0.8 mm and 3.2 ± 0.8 mm, comparable with those in our study group, confirming that the screw-drilling guidance is important to enhancing the accuracy of reconstruction.^13^

In evaluating the accuracy of bone reconstruction, we delineated and compared the center point and central axis of each bone graft, similar to the method in the Schepers et al. study using CNC-milled patient-specific surgical plates. In the seven cases studied by Schepers et al.,^26^ the distance deviation of fibular bone grafts was 2.9 ± 1.7 mm, and the angulation deviation was 4.3 ± 3.2°, approximating our results for our study group.^26^ The inter-operator agreement for reconstruction measurements in this study was at least good, indicating the high reliability of the measured parameters.^27^

With the advancement of microvascular surgery, the survival rates for vascularized bone flaps have been ultimately improved, and surgeons are pursuing more accurate reconstruction results for enhancement of aesthetic and functional outcomes. It is well-known that bone deviations may severely affect factors such as aesthetics, occlusion, and condylar positions, highlighting the clinical significance of improved reconstruction accuracy. However, it is a concerning question whether the quantitative accuracy is clinically relevant or not.

On the one hand, the 3D-printed patient-specific plates improved the accuracy of reconstruction by one-third in the entire maxilla or mandible, by one half in the distance deviation of the bilateral condylar heads, and by nearly three-fourths in the distance deviation of the bilateral mandibular angles compared with conventional plates. The improved accuracy definitely is clinically significant for enhancement of aesthetics, occlusion, and condylar functions. Based on the increased accuracy, we currently have started simultaneous dental implantation during surgery, which further pushes forward this exciting new frontier.

On the other hand, the small inaccuracy with hard tissue can be covered by soft tissue in aesthetics. Meanwhile, the mobile mandible also can accommodate slight malocclusion and minor joint dislocation. Knowledge of the minimal clinically important difference (MCID) in head and neck reconstruction still is lacking.^1^ Therefore, the relationship between quantitative accuracy and clinical outcomes remains to be elucidated, and the clinical relevance of our results cannot be overinterpreted.

The lack of MCID in head and neck reconstruction further highlights the importance of the current quantitative study. The measured outcomes in the current study may provide a good reference for future studies to clarify the clinically relevant accuracy in head and neck reconstruction.

In addition to the clinical significance, the improved accuracy endowed by 3D-printed patient-specific surgical plates is particularly important for the continuing progress of head and neck reconstruction because it enhances the predictability and repeatability of accurate head and neck reconstruction to ensure quality control of surgery, especially for young surgeons and residents.^6^ For example, the 3D-printed patient-specific surgical plates can cope with more challenging cases.^28^–^30^

In the current study, more patients in the study group had reconstruction with the “double-barrel” fibula flaps, which usually are too complicated to be arranged accurately by conventional plates considering the multiple bone segments.^31^ However, with the 3D-printed patient-specific surgical plates, all bone segments can be easily arranged and secured. This underscores the importance of technological advancements of patient-specific surgical plates in head and neck reconstruction.

The accuracy of osteotomy also was evaluated. The basic principle of cutting guides is to direct the exact position and direction of osteotomy, so we delineated the osteotomy planes and located corresponding center points for accuracy analysis.^21^–^23^ In the study by Maesschalck et al.^32^, the distance deviations of osteotomy were 2.3 ± 1.0 mm in the mandible and 1.9 ± 1.1 mm in the fibula, comparable with our results. As expected, we found no significant difference in osteotomy between the two groups because both groups used cutting guides for osteotomy, confirming that the improved accuracy of reconstruction is induced by the 3D-printed patient-specific surgical plates.

From our experience, application of 3D-printed patient-specific surgical plates facilitated the surgical manipulation and reduced the operative time, as reported in other studies.^28^,^33^,^34^ However, in the current study, these parameters did not differ significantly between the study and control groups. However, these results need to be interpreted with caution. These end points are significantly affected by multiple confounding factors, especially comorbidities of the patients, extension of the tumors, and complexity of the surgery.^35^

For further verification of these outcomes, more patients with well-controlled confounding factors should be recruited. Furthermore, our previous study indicated that CAS saves time and increases efficiency compared with conventional freehand surgery.^36^ Therefore, the marginal increased efficiency of the 3D-printed patient-specific surgical plates may not have been well reflected, especially considering that the number of patients was small in this study.

Another issue calling for discussion is the cost-effectiveness, mechanical strength, and accessibility of the 3D-printed patient-specific surgical plates.^1^ Because medical treatment in our Hong Kong public hospital is supported primarily by the government, the current study had no cost-effectiveness analysis. Due to the high cost of CAS and 3D-printing technology in other countries or areas, the wide application of 3D-printed patient-specific surgical plates may be restricted. In our study, the additive manufacturing of titanium was performed using the SLM technology. According to our knowledge, the yield strength, tensile strength, elastic modulus, and Poisson’s ratio of melted titanium plates are similar to those of forged plates. However, the elongation properties are poorer, which should be confirmed and improved in additional studies.

Concerning the accessibility of SLM, with our proposed “surgeon-dominated” workflow, surgeons are encouraged to design patient-specific surgical plates, whereas expert engineers are responsible for the optimization and production.^6^ The titanium SLM is explosive and should be performed with special equipment under an argon atmosphere, which cannot be completed without industrial support.^6^ However, the enhanced reconstruction accuracy, as shown in the current study, supports the rationale for applying 3D-printed patient-specific surgical plates in head and neck reconstruction, which likely would promote the development and generalization of this new technology and reduce the cost in the near future.

Certain limitations of the present study need to be addressed. First, our study was conducted by the same chief surgeon, thus precluding the potential biases incurred with multiple surgical teams that may have varied surgical experience and preferences.^37^ Second, the follow-up periods differed between the study and control groups. In evaluating the accuracy of reconstruction, we recommend that follow-up CT scans be collected within 3 months after surgery, as in the study group.^24^ However, with more accurate restoration of anatomic structures using CAS, we predict that only minor changes would occur in the long term, which should not jeopardize the main results in this study. The longitudinal changes in maxilla or mandible reconstruction should be investigated in future studies for a comparison of both the short- and long-term outcomes.

Third, although it was reasonable to investigate the accuracy of head and neck reconstruction by including both mandible and maxilla patients, we acknowledge that the sample was too small for a compelling subgroup analysis. However, our results still were sufficient to show the increased accuracy provided by patient-specific surgical plates. Especially considering the novelty and relatively the few studies analyzing patient-specific plates, our study may promote more research and provide a good reference for future studies.

In conclusion, this is the first study to prove that compared with conventional plates, 3D-printed patient-specific surgical plates can precisely transfer a virtual surgical plan to real surgery, leading to enhanced accuracy of oncologic head and neck reconstruction. This quantitative study constitutes the highest level of evidence to date. The increased prevalence 3D-printed patient-specific surgical plates used in clinical practice should lead to well-designed prospective clinical trials to confirm our results in the near future.

## Electronic supplementary material

Below is the link to the electronic supplementary material.

Supplementary material 1 (DOCX 522 kb)
