# Supplementary material for: Three-Dimensionally Printed Patient-Specific Surgical Plates Increase Accuracy of Oncologic Head and Neck Reconstruction Versus Conventional Surgical Plates: A Comparative Study
Source: Ann Surg Oncol. 2020 Jun 22;28(1):363–75. doi: 10.1245/s10434-020-08732-y (PMC7752789; doi:10.1245/s10434-020-08732-y)
Supplement: Supplementary file 1 — Supplementary material 1 (DOCX 522 kb) [file 10434_2020_8732_MOESM1_ESM.docx]

*Supplemental Files*

**Three-Dimensional Printed Patient-Specific Surgical Plates Increase Accuracy of Oncological Head and Neck Reconstruction Versus Conventional Surgical Plates: A Comparative Study**

Wei-fa Yang, MDS^1#^; Wing Shan Choi, MDS^1#^; May CM Wong, PhD^2^; Warit Powcharoen, DDS^3^; Wang-yong Zhu, MD^1^; James Kit-Hon Tsoi, PhD^4^; Marco Chow, MPhil^5^; Ka-Wai Kwok, PhD^5;^ Yu-xiong Su, MD^1*^.


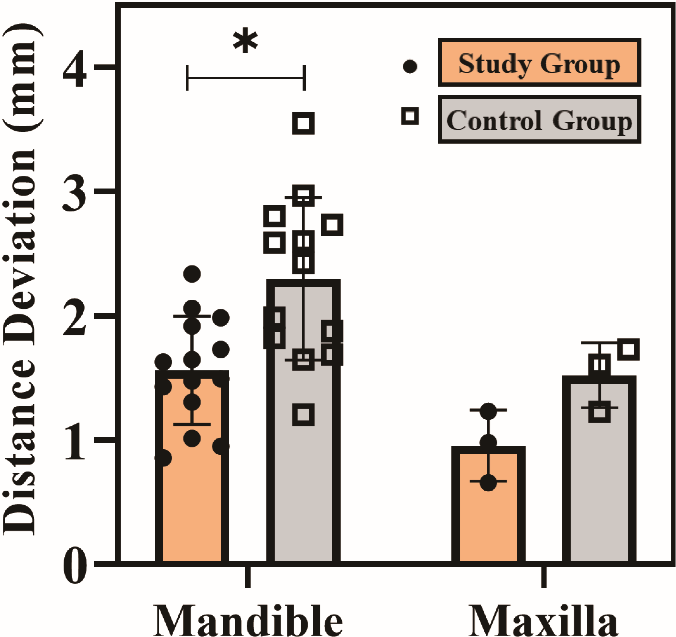


**Supplemental Figure 1**. Mean absolute distance deviation of the integral mandible or maxilla. Either in mandibular or maxillary reconstruction, additively manufactured patient-specific surgical plates showed superior accuracy results versus the control group of conventional plates. Due to the small number of cases (n=3) in maxillary reconstruction, a significant difference is not attained. In all bars, the mean values with standard deviations are depicted. *p < 0.05.


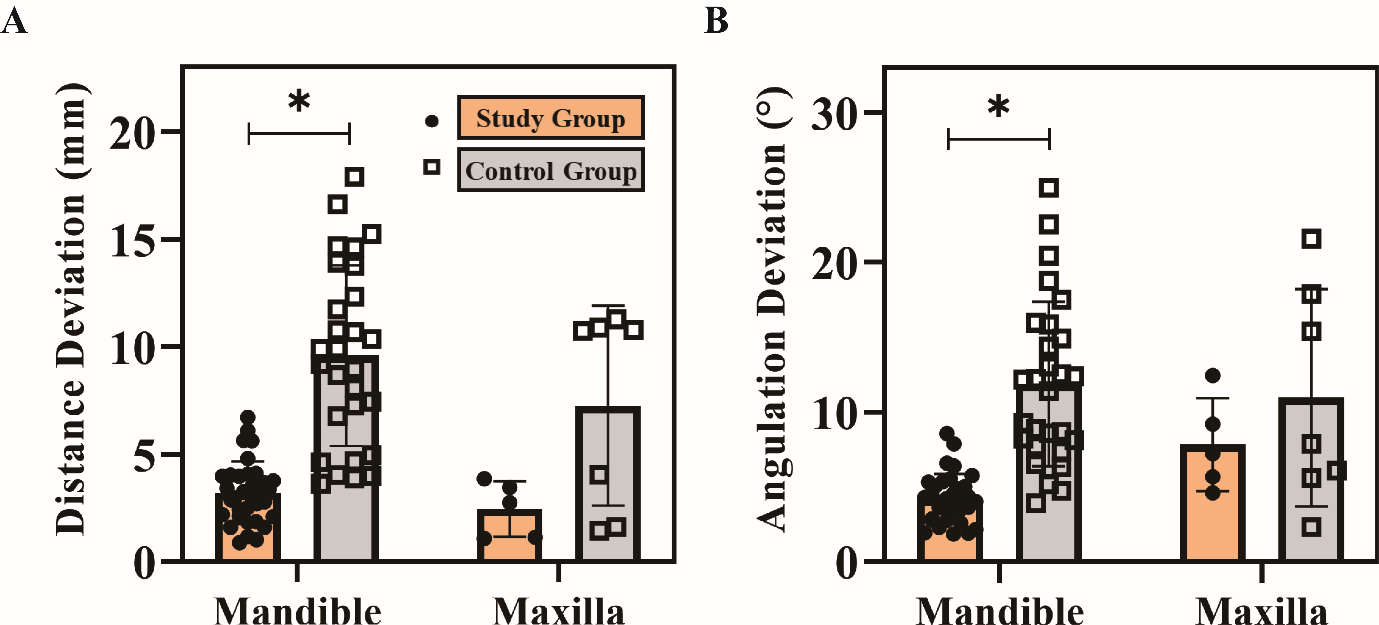


**Supplemental Figure 2**. Accuracy of reconstructed bone grafts. **(A)** Distance deviations of reconstructed bone grafts. **(B)** Angulation deviations of reconstructed bone grafts. In all bars, the mean values with standard deviations are depicted. *p < 0.05.


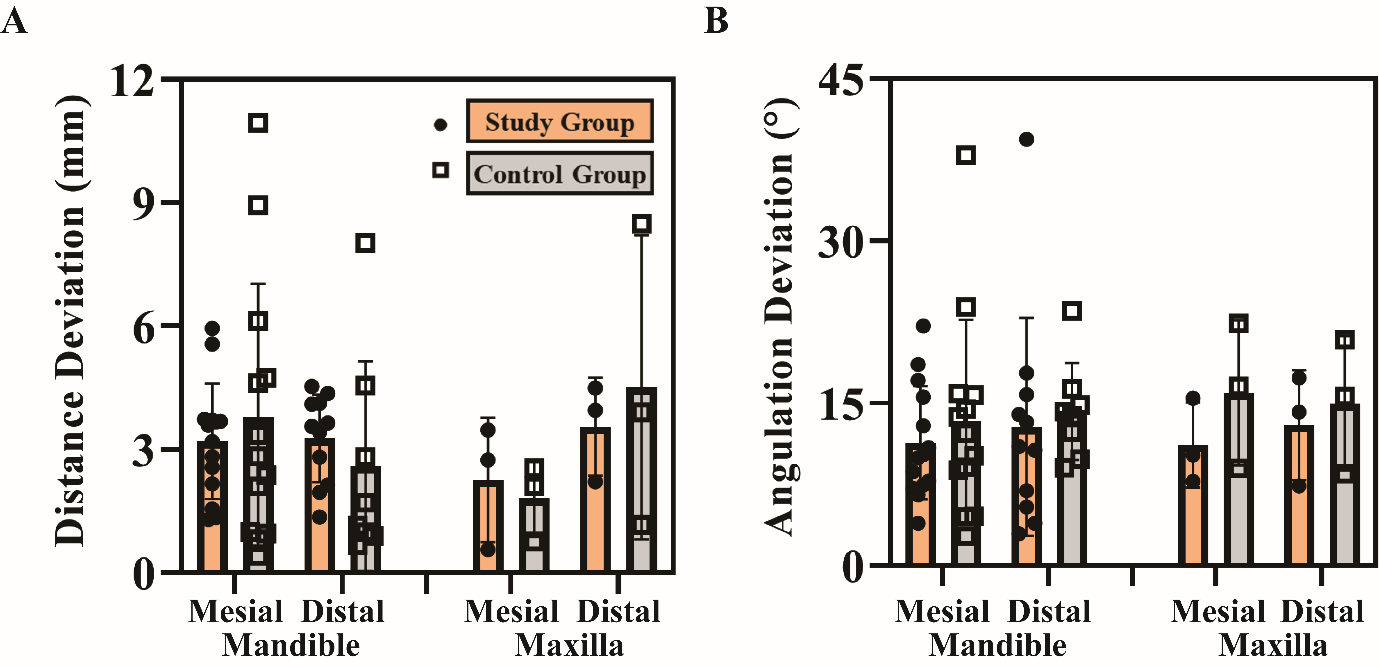


**Supplemental Figure 3.** Accuracy of skull bone resection. **(A)** Distance deviations of maxillary and mandibular resections assisted by cutting guides, either in mesial or distal sites. **(B)** Angulation deviations of maxillary and mandibular resections, either in mesial or distal sites. No significant difference in the accuracy of skull bone resection is found. In all bars, the mean values with standard deviations are depicted.

**Supplemental Table 1. Inter-Operator Agreement in Accuracy Analysis of Reconstruction and Osteotomy.**

| **Variables** | **Interrater Reliability** | **Reliability** |
| --- | --- | --- |
| **Reconstruction** |  |  |
| Integral maxilla/mandible |  |  |
| Distance Deviation | 0.780 (0.480, 0.899) | Good |
| Condylion |  |  |
| Distance Deviation | 0.994 (0.988, 0.997) | Excellent |
| Angulation Deviation | 0.987 (0.973, 0.994) | Excellent |
| Gonion |  |  |
| Distance Deviation | 0.997 (0.993, 0.999) | Excellent |
| Angulation Deviation | 0.981 (0.959, 0.991) | Excellent |
| Bone graft |  |  |
| Distance Deviation | 0.937 (0.897, 0.961) | Excellent |
| Angulation Deviation | 0.884 (0.816, 0.927) | Excellent |
| **Osteotomy** |  |  |
| Maxilla/mandible |  |  |
| Distance Deviation | 0.726 (0.537, 0.838) | Good |
| Angulation Deviation | 0.619 (0.355, 0.775) | Fair |
| Bone graft |  |  |
| Distance Deviation | 0.758 (0.618, 0.841) | Good |
| Angulation Deviation | 0.604 (0.452, 0.714) | Fair |

*a*. Data are means and 95% CI.

*b*. The level of inter-operator agreement is considered excellent, good, fair, and poor when ICC>0.81, 0.80>ICC>0.71, 0.70>ICC>0.51, and 0.50>ICC, respectively.

**Supplemental Table 2. Demographics and Clinical Information of Patients**

| **Case** | **Sex** | **Age**  **(yr)** | **Lesion Site** | **Diseases** | **pTNM*** | **Bone Graft** |
| --- | --- | --- | --- | --- | --- | --- |
| **Study Group (n=17)**  **3D-printed Patient-specific Surgical Plates** | | | | | | |
| 1 | F | 69 | Left mandible | Squamous cell carcinoma | T2N0M0 | Fibula |
| 2 | F | 66 | Left maxilla | Osteosarcoma | T1N0M0G3 | Fibula |
| 3 | F | 58 | Right maxilla | Squamous cell carcinoma | T3N2aM0 | Fibula |
| 4 | F | 57 | Right mandible | Squamous cell carcinoma | T3N3bM0 | Iliac crest |
| 5 | F | 33 | Anterior maxilla | Ameloblastoma | NA | Fibula |
| 6 | F | 54 | Left mandible | Squamous cell carcinoma | T4aN0M0 | Fibula |
| 7 | F | 64 | Right mouth floor | Squamous cell carcinoma | T4aN1M0 | Fibula |
| 8 | F | 75 | Left mandible | Secondary defect | NA | Fibula |
| 9 | M | 22 | Left mandible | Ameloblastoma | NA | Fibula |
| 10 | F | 55 | Left mandible | Squamous cell carcinoma | T3N2aM0 | Iliac crest |
| 11 | F | 35 | Right Mandible | Ameloblastoma | NA | Fibula |
| 12 | M | 64 | Right Mandible | Squamous cell carcinoma | T2N0M0 | Fibula |
| 13 | M | 67 | Anterior Mandible | Squamous cell carcinoma | T2N0M0 | Fibula |
| 14 | F | 65 | Right Mandible | Squamous cell carcinoma | T4aN0M0 | Fibula |
| 15 | F | 44 | Right Mandible | Squamous cell carcinoma | T2N0M0 | Fibula |
| 16 | M | 52 | Left Mandible | Mesenchymal chondrosarcoma | NA | Fibula |
| 17 | F | 65 | Left Mandible | Osteoradionecrosis | NA | Fibula |
| **Control Group (n=16)**  **Conventional Surgical Plates** | | | | | | |
| 1 | F | 42 | Right maxilla | Malignant histiocytosis | NA | Fibula |
| 2 | F | 39 | Left mandible | Squamous cell carcinoma | T4aN0M0 | Fibula |
| 3 | F | 72 | Left mandible | Squamous cell carcinoma | T2N0M0 | FIbula |
| 4 | F | 49 | Left mandible | Secondary defect | NA | Fibula |
| 5 | F | 54 | Rigth mandible | Squamous cell carcinoma | TisN0M0 | Fibula |
| 6 | F | 55 | Left maxilla | Ameloblastic carcinoma | T4aN0M0 | Fibula |
| 7 | F | 38 | Left mandible | Ameloblastoma | NA | Fibula |
| 8 | F | 22 | Left mandible | Benign nerve sheath tumor | NA | Fibula |
| 9 | F | 67 | Left mandible | Ameloblastoma | NA | Fibula |
| 10 | M | 74 | Right mandible | Squamous cell carcinoma | T4aN0M0 | Fibula |
| 11 | M | 81 | Left mandible | Squamous cell carcinoma | T4aN2bM0 | Fibula |
| 12 | M | 40 | Left mandible | Ameloblastic fibroma | NA | Iliac crest |
| 13 | M | 67 | Right mandible | Squamous cell carcinoma | T3N0M0 | Fibula |
| 14 | M | 59 | Right mandible | Adenoid cystic carcinoma | T4N2aM0 | Fibula |
| 15 | F | 61 | Right maxilla | Squamous cell carcinoma | T2N0M0 | Fibula |
| 16 | F | 66 | Right mandible | Squamous cell carcinoma | T1N0M0 | Fibula |

Abbreviations: yr, year; NA, not applicable.

*According to the AJCC (American Joint Committee on Cancer) Cancer Staging Manual (8th Edition).
